# Fetal Magnetic Resonance Imaging in Association With Antenatal Ultrasound in the Diagnosis of Caudal Dysgenesis: Report of Two Cases

**DOI:** 10.7759/cureus.35485

**Published:** 2023-02-26

**Authors:** Mohamed H Mahmoud, Taymoor Asghar, Tarek H Elkammash, Ahmed M Housseini, Azza A Gad

**Affiliations:** 1 Radiology, Faculty of Medicine, Suez Canal University, Ismailia, EGY; 2 Radiology, Sheffield University Hospital, Sheffield, GBR; 3 Radiology, Suez Canal University Hospital, Ismailia, EGY

**Keywords:** antenatal ultrasound, foetal mri, prenatal diagnosis, sacral agenesis, caudal regression syndrome

## Abstract

Caudal regression syndrome is a relatively rare congenital disorder that consists of a constellation of caudal developmental growth abnormalities and associated soft tissue anomalies. The severity of its spectrum ranges from lumbosacral agenesis to isolated absent coccyx. We report two cases of caudal regression syndrome that were diagnosed in utero at different gestational ages by prenatal ultrasound followed by fetal MRI for a complete assessment of the associated imaging features. When used in association with antenatal ultrasonography, fetal MRI is particularly instructive in the prenatal diagnosis of caudal regression syndrome since it overcomes the limits of obstetric ultrasound, provides additional information, including associated local soft tissue abnormalities and manifestations of syndromic processes, and allows for a more accurate evaluation of the spinal cord.

## Introduction

Caudal regression syndrome is defined as an abnormally formed lumbosacral vertebral column due to abnormal notochord formation in utero, frequently associated with spinal cord abnormalities as well as anorectal and genitourinary malformations [1]. Caudal regression syndrome is classified into two types, with L1 being the discriminatory level in relation to the position of the conus medullaris: in type 1, there is a hypoplasia of the distal spinal cord with associated marked sacral osseous anomalies, while in type 2 there is tapered, low-lying spinal cord with distal cord elongation and tethering, but less marked sacral anomalies [2]. The spectrum of caudal regression syndrome can range from sacral agenesis or dysgenesis to sirenomelia (lower limbs fusion) [3]. The presence of maternal gestational diabetes increases the incidence of caudal regression 200-fold from the baseline of 0.1 to 0.25 per 10,000 normal pregnancies [2].

Caudal regression can be isolated or associated with other congenital anomalies like omphalocele, exstrophy of the urinary bladder, imperforate anus, and spinal defects (OEIS complex); vertebral abnormalities, anal anomalies, cardiac anomalies, tracheoesophageal fistula, limb malformations, hydrocephalus (VACTERL-H); and sacral agenesis, anal atresia, and sacrococcygeal teratoma or meningocele (Currarino triad) [4].

Antenatal sonographic findings of caudal regression syndrome are variable. There may be a complete absence of the sacral vertebrae with associated lumbar spine and lower limbs abnormalities, including club feet, hip, and knee contractures. Non-visualization of several vertebrae and fusion of the iliac bones can also be noted. A transverse ultrasound view of the fetal abdomen may fail to show the normal spine. It is important to note that sacral spine ossification centers are first visible at 16-17 weeks of gestation, and therefore one must be careful to avoid falsely calling sacral dysgenesis in early scans [4]. Decreased fetal lower limb movements during the examination may be present. An abnormally increased amniotic fluid index has been reported (polyhydramnios), although this is likely due to the association with gestational diabetes rather than sacral dysgenesis itself [5-7].

Fetal MRI can both determine the level of conus medullaris and confirm the diagnosis of caudal regression syndrome. Both axial and sagittal MRI views are helpful in assessing the number of sacral segments. When there is decreased amniotic fluid index (oligohydramnios), fetal MRI is superior to antenatal ultrasound in the assessment of associated fetal anomalies [2]. When only a few sacral segments are absent, the diagnosis of caudal regression syndrome can be challenging both by US and MRI. In mild cases of caudal regression syndrome, only the tip of the conus medullaris is absent and cannot be detected antenatally [2].

We report two cases of caudal regression syndrome diagnosed by antenatal fetal ultrasound and MRI at different gestational ages.

## Case presentation

Case 1

**Figure 1:**
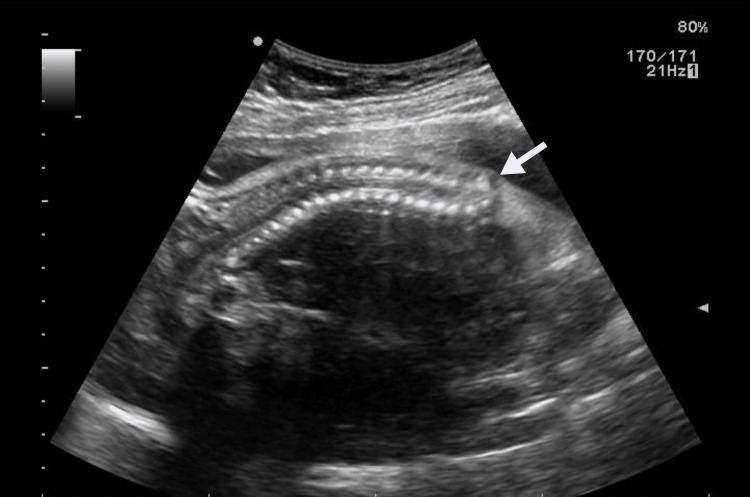
Antenatal ultrasound-midsagittal view shows abrupt termination of the spine at the level of the lumbar spine with an absent sacrum (arrow)

**Figure 2:**
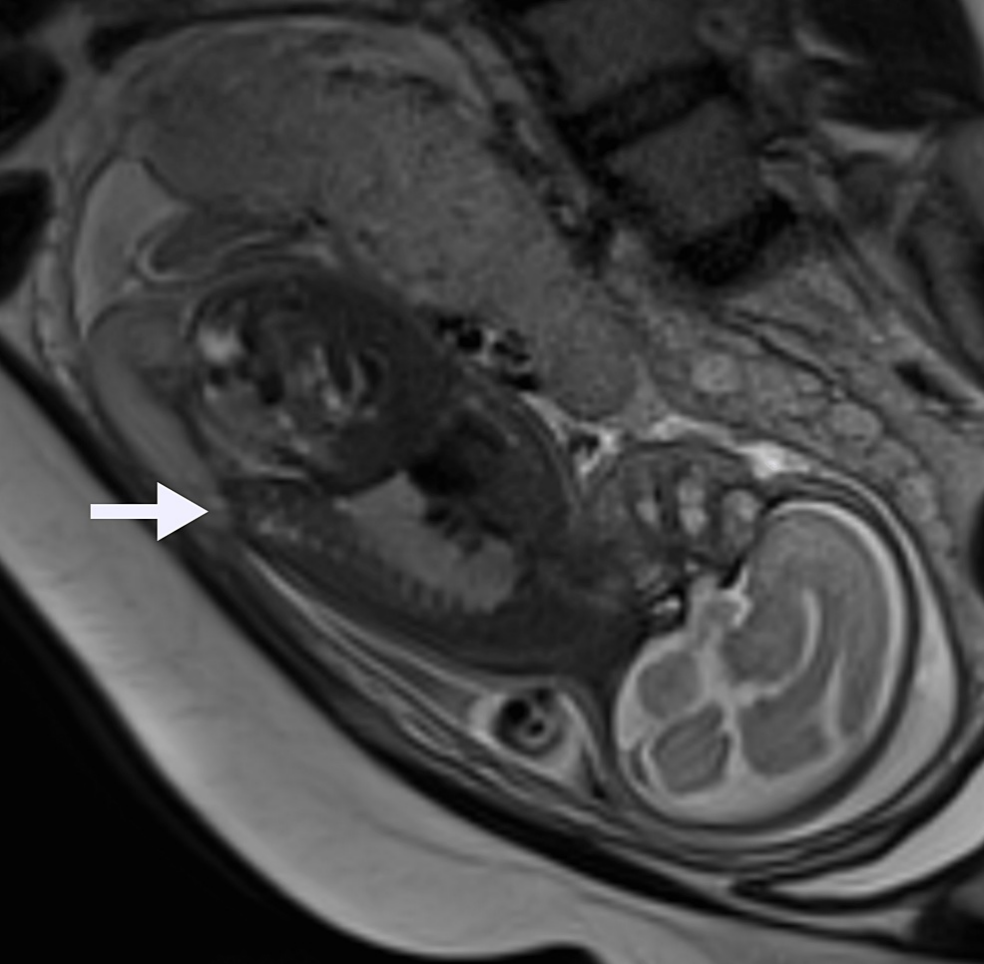
Antenatal sagittal magnetic resonance T2-weighted image shows abrupt termination of the spine at lumbar level (arrow)

**Figure 3:**
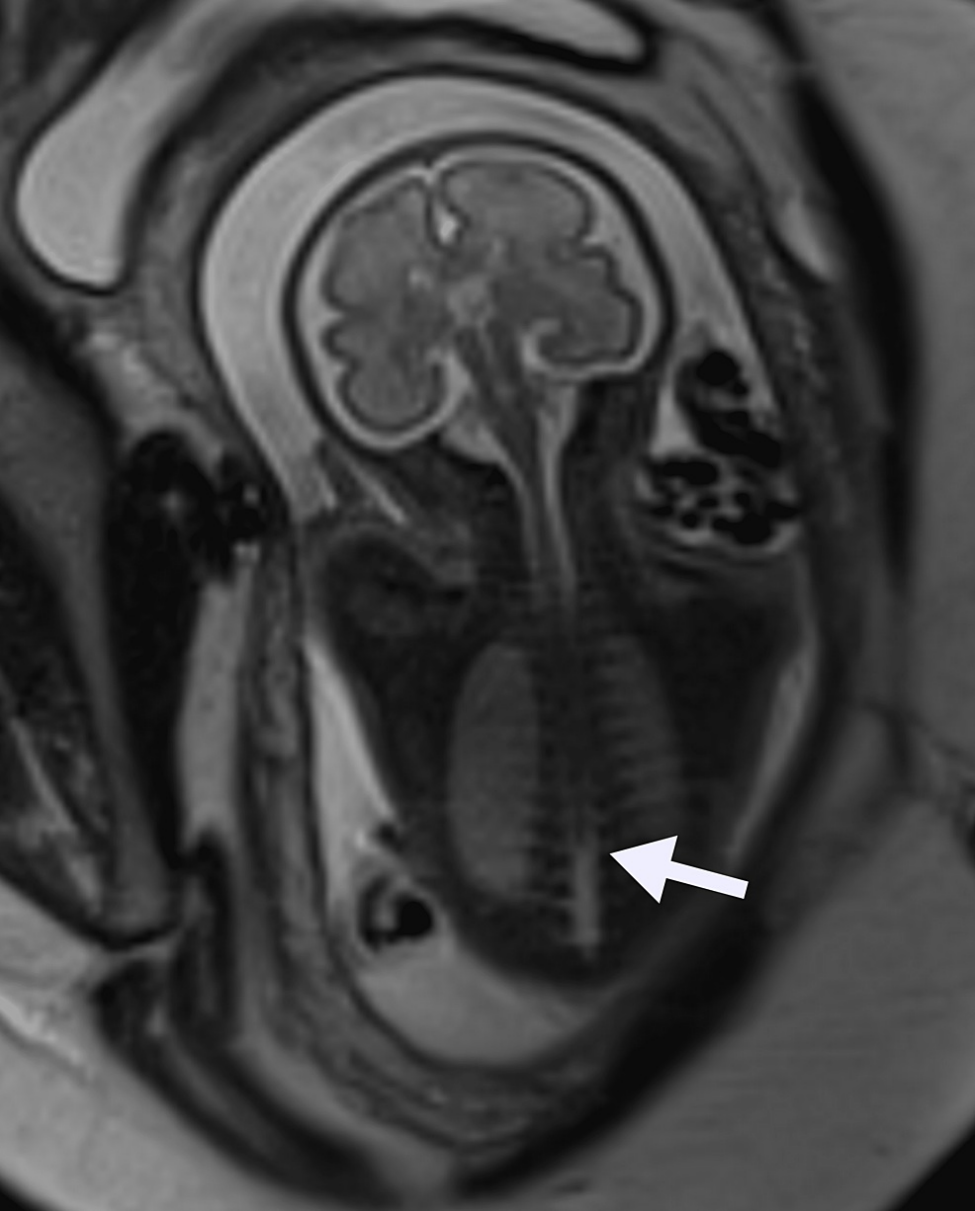
Antenatal coronal magnetic resonance T2-weighted image showing the spinal cord ending at the mid-thoracic level (arrow)

A 22-year-old primigravida with no personal or first-degree family history of chronic illness presented at 24 weeks +3 days of gestation for a routine antenatal visit and underwent an ultrasound examination which showed a single viable fetus with fetal spine abnormalities seen as abrupt termination of the lumbar spine with absent sacrum raising the possibility of sacral agenesis (Figure 1). A fetal MRI was performed at 28 weeks of gestation to confirm the diagnosis of caudal regression syndrome and for screening of other abnormalities. Fetal MRI showed the bony elements of the fetal spine stop at T12/L1 level (Figure 2). The spinal cord stops at the mid-thoracic level (Figure 3); however, no bony component of the lumbar or sacral spine with a hypoplastic bony pelvis and a lack of muscle around the abdomen and pelvis. No other abnormalities were found.

**Figure 4:**
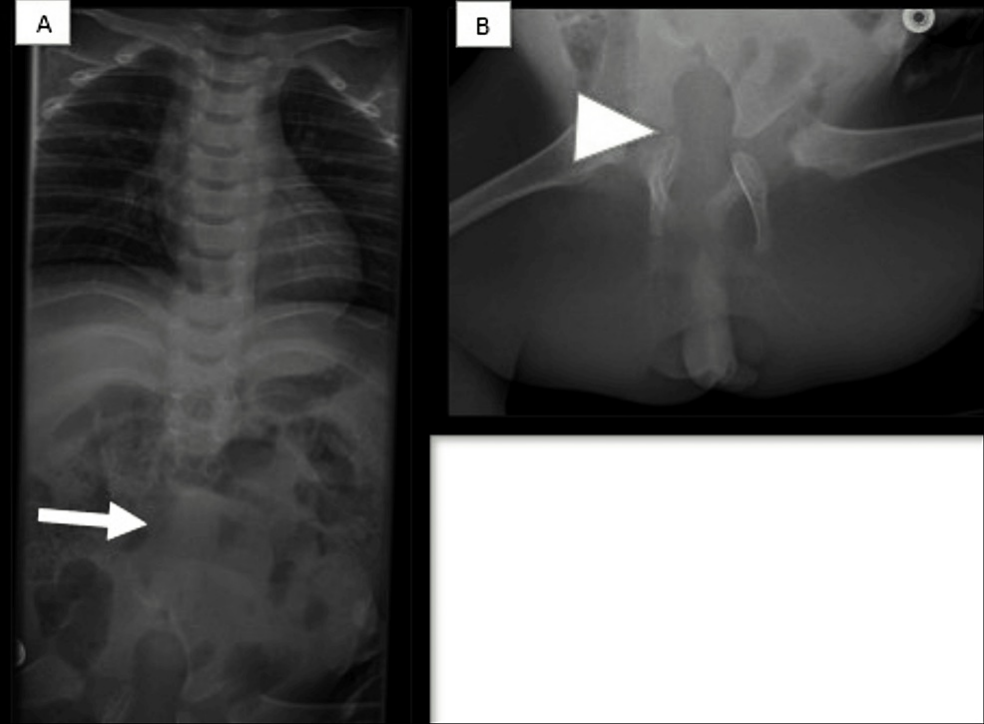
Post-natal X-Ray-AP views (A) Post-natal X-Ray-AP view shows absent lower lumbar spine, sacral spine, and coccyx (arrow) with the hypoplastic pelvis. (B) Post-natal X-Ray-AP view shows a hypoplastic pelvis with a very narrow eight-shaped pelvic inlet (arrowhead).

**Figure 5:**
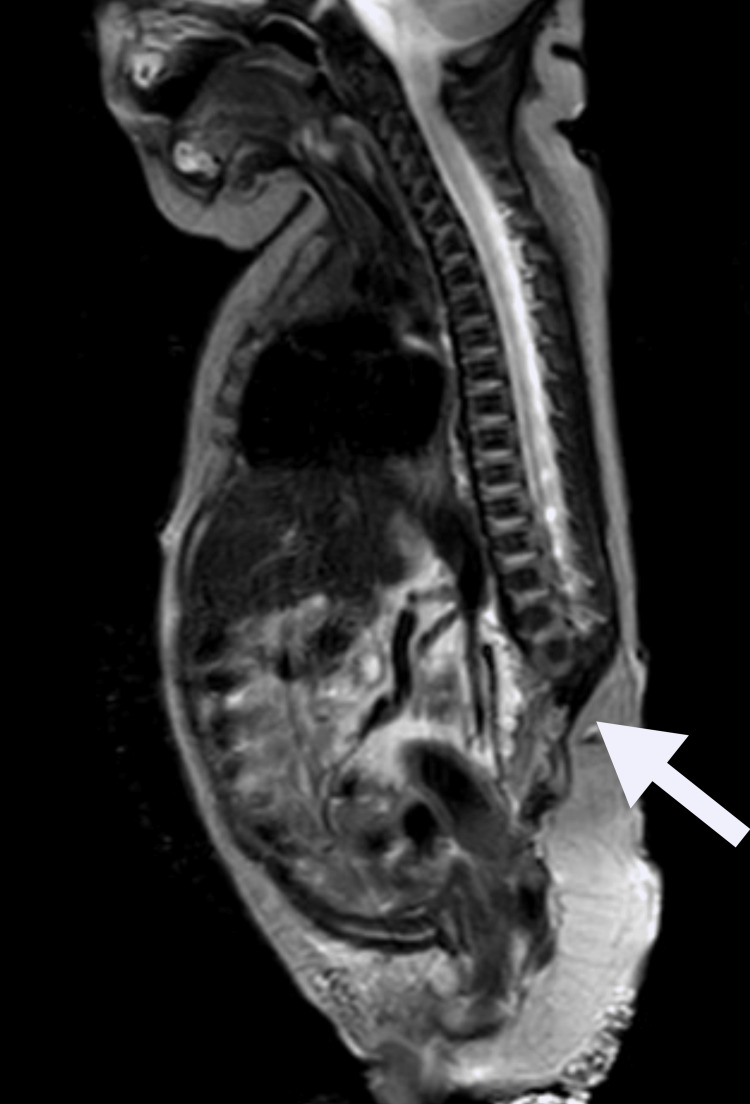
Post-natal magnetic resonance T2-weighted image shows absent lower lumbar spine, sacral spine, and coccyx (arrow)

Post-natal skeletal survey showed the presence of 12 pairs of ribs and one ossified lumbar vertebral body. The remainder of the lumbar and sacral spine and coccyx are not ossified, with abnormal modeling of the pelvis and a very narrow eight-shaped pelvic inlet (Figure 4). The radiographic features are those of caudal regression syndrome, which confirms the MRI findings. Post-natal MRI (Figure 5) was also performed and confirmed the diagnosis of caudal regression syndrome with high-ending conus medullaris and severe sacral osseous abnormality in keeping with type 1 caudal regression syndrome.

Case 2

**Figure 6:**
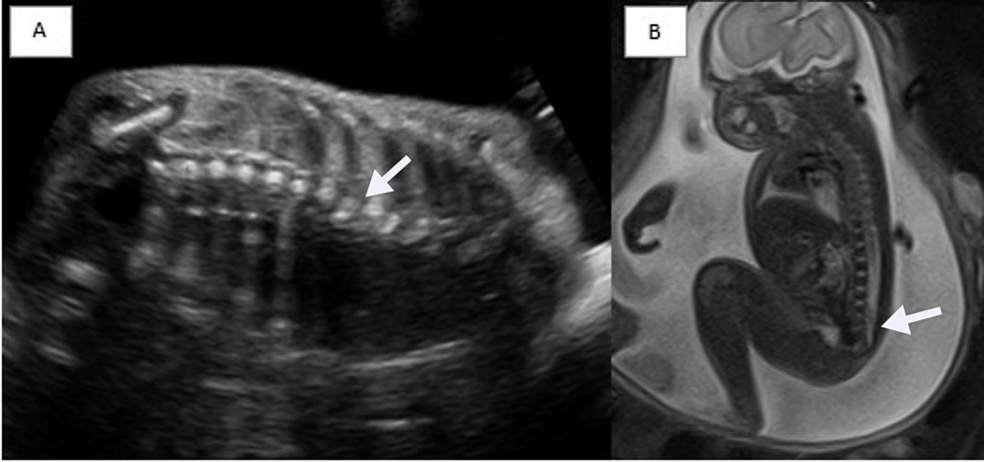
Antenatal ultrasound and fetal magnetic resonance T2-weighted image (A) Antenatal ultrasound (longitudinal view) shows mild malalignment of the lumbar spine. (B) Fetal magnetic resonance T2-weighted image sagittal view shows an absent sacral spine with absent normal lumbar lordosis. The canal appears wide and ends abruptly, raising the suggestion of sacral agenesis.

A 25-year-old primigravida with no personal or family history of chronic illness was referred by the obstetrician for fetal MRI as the screening antenatal ultrasound scan performed at 29 weeks of gestation showed a malaligned spine, ventricular septal defect (VSD), and two vessels umbilical cord. Fetal MRI showed mild kyphosis at the mid-thoracic region with normal spinal cord throughout the thoracic and upper lumbar spine. The spinal cord terminates just below the level of the fetal kidneys. The lumbar spine appears normal; however, the sacral spine was not clearly seen, and the normal lumbar lordosis was absent. The canal appears wide and ends abruptly, raising the suggestion of sacral agenesis (Figure 6). The conus medullaris was seen elongated at extending below the level of the L1 vertebra. No other abnormalities were detected. The presence of a mild form of sacral agenesis was confirmed postnatally.

## Discussion

Caudal regression syndrome is a rare congenital disorder. It is classified into two main types based on the shape and location of the conus medullaris and the associated sacral abnormalities [2]. Type 1 caudal regression syndrome is characterized by distal cord hypoplasia that is blunted and terminates abruptly proximal to the level of L1. Marked sacral anomalies, in addition to urinary bladder dysfunction, are more often in type 1 than in type 2. Type 2 caudal regression syndrome is characterized by an abnormally low location of conus medullaris that ends distal to the first lumbar vertebra, and it is often elongated and tethered. Less severe sacral anomalies are associated with type 2.

Caudal regression syndrome can be diagnosed in the second or third trimester with antenatal ultrasound, which shows sacrococcygeal dysgenesis with a high and abrupt termination of the spine at the lumbosacral level.

For most exams, ultrasonography can be used to identify the spinal cord within the spinal canal. However, it is obscured to a varying extent by the existence of several spinal ossification centers, particularly later in gestation. The spinal cord appears homogeneous on ultrasound, progressively getting smaller from cranial to caudal, terminating as the conus medullaris. Spina bifida occulta and tethered cord can both be identified by measuring the distance between the conus medullaris and the tip of the spine [2],

Severe forms of the syndrome can also be suspected as early as the end of the first trimester on observation of a short crown-rump length and abnormal posture of lower limbs (usually contractures) [2], although this must be confirmed with a second-trimester scan after vertebral ossification to avoid false positives. Ultrasound is also a useful tool in the assessment of any other associated abnormalities, such as renal anomalies and gastrointestinal anomalies [8]. Fetal MRI helps to confirm the diagnosis of caudal regression syndrome and, more importantly, to identify the level of the conus medullaris, in which aspect it is superior to ultrasound. Cine sequences are sometimes helpful in raising suspicion of the diagnosis on observation of limited lower limb mobility and contractures.

Especially in the setting of decreased amniotic fluid (oligohydramnios) and maternal obesity, MRI is a helpful tool that can be used for the assessment of any associated brain, genitourinary, gastrointestinal, and musculoskeletal abnormalities [9]. A wide field of view localizer can document fetal lie, placental location, and amniotic fluid volume. In order to account for fetal motion, subsequent imaging sequences with thinner slices and a smaller field of view are recommended in orthogonal planes to the fetal spine, brain, and other suspected abnormalities [4]. Thin-cut (2-4 mm) T2-weighted images (single-shot fast spin-echo [SSFSE] or half-Fourier acquisition single-shot turbo spin-echo [HASTE] sequences) are valuable in the assessment of spinal/cranial abnormalities. Fast T1-weighted gradient-echo imaging is helpful in the evaluation of fat and blood content. Rapid gradient-echo, echo-planar imaging (EPI - fetal black bone) can be suitable for vascular and skeletal compartment delineation [4].

In the first case, we represent typical radiological findings in type 1 caudal regression syndrome. The ultrasound showed an abrupt ending of the lumber spine with non-visualization of the sacral spine. Additionally, fetal MRI showed abrupt termination of the spinal cord in the mid-thoracic level with the termination of the spinal cord at the level of L1, hypoplastic pelvis, and lack of muscles around the pelvis. All these described imaging features are consistent with the diagnosis of type 1 caudal regression syndrome. The first case demonstrates the importance of fetal MRI in the assessment of the spinal cord, level of conus medullaris, and fetal musculature.

In the second case, we present typical radiological findings in type 2 caudal regression syndrome. Initially, the antenatal ultrasound showed a malaligned spine in addition to other abnormalities such as VSD and two vessels cord. By fetal MRI, the lumbar spine appears normal; however, the sacral spine was not visualized with absent normal lumbar lordosis. Additionally, the canal appears wide and ends abruptly, and the conus medullaris was seen elongated and extending below the level of the L1 vertebra. The described imaging features are in keeping with type 2 caudal regression syndrome with absent distal sacral segments and low-lying conus medullaris.

## Conclusions

Antenatal ultrasonography is considered the initial modality for diagnosing and detecting cases of caudal regression syndrome. Fetal MRI is helpful in the prenatal diagnosis of caudal regression syndrome when combined with antenatal ultrasound since it overcomes the drawbacks of ultrasound, such as poor image quality in the presence of oligohydramnios and maternal obesity, and provides additional details and a more accurate evaluation of the spinal cord.
